# Varied Presentation of Oropharyngeal Tuberculosis: Review of Cases

**DOI:** 10.7759/cureus.43636

**Published:** 2023-08-17

**Authors:** Shaila Sidam, Anjan K Sahoo, Vikas Gupta, Ujjawal Khurana

**Affiliations:** 1 Otolaryngology - Head and Neck Surgery, All India Institute of Medical Sciences, Bhopal, IND; 2 Otorhinolaryngology, All India Institute of Medical Sciences, Bhopal, IND; 3 Pathology and Laboratory Medicine, All India Institute of Medical Sciences, Bhopal, IND

**Keywords:** tuberculosis, tonsillitis, pott’s spine, oropharyngeal, abscess

## Abstract

Tuberculosis (TB) is a chronic granulomatous infectious disease with 25% morbidity due to extrapulmonary form. Hence, knowledge about varied presentations of extrapulmonary oropharyngeal type may help in early diagnosis and management in acute as well as chronic settings.

This article describes immunocompetent patients’ presentation with varied oropharyngeal manifestations and later diagnosed with tuberculous tonsillitis and tuberculous abscesses with Pott’s spine.

The varied manifestation of oropharyngeal TB, which is supposed to be a chronic condition, may help in early diagnosis in acute and chronic settings.

## Introduction

The extrapulmonary form of tuberculosis (TB) accounts for 25% of all tuberculous morbidity. As per WHO (Global TB Report 2021), an estimated 10 million people fall ill yearly due to TB, and 1.5 million die [1] One in every five patients of TB presents with extrapulmonary TB. The commonest site to be affected is the lymph node followed by other sites such as the pleura, skeletal system, CNS, abdomen, and genitourinary tract [2] Cervical lymphadenopathy is the commonest form of manifestation of TB in ENT apart from otitis media, laryngitis, pharyngitis, and nasal TB which constitute significantly lesser variants [3]. There is an increase in incidence because of immunodeficient survivors, because of the emergence of drug-resistant strains and the HIV epidemic creating a global health crisis. The diagnosis is difficult because according to the organ affected, clinical and radiological features will depend on which can mimic other diseases, and, hence, the diagnosis is based on clinical, radiological, bacteriological, and pathological examination. The diagnosis is confirmed by cytopathology or histopathology along with or without ZN staining and mycobacterial culture. Here, we report cases of oropharyngeal TB without pulmonary focus. The aim is to create awareness about the varied presentation of oropharyngeal TB, so as to consider it in differential diagnosis of atypical oropharyngeal lesions.

## Case presentation

Case 1

A 26-year-old male presented to the outpatient department with complaints of pain in the throat and difficulty in swallowing for 10 days. In addition, he complained of swelling in the left side of his neck for one and a half months. He had a history of addiction to chewing tobacco for four to five years. On examination of the oral cavity, there was left tonsillar hypertrophy, and there was cervical lymphadenopathy on the left side of the neck.

The fine needle aspiration cytology report from the left cervical lymph node showed signs of granulomatous lymphadenitis, and from the left tonsil, it was tuberculous tonsillitis. ZN stain was positive. The Hb was 13 g/dl, WBC was 9000/µl, and ESR was 16 mm at the end of the first hour.

**Figure 1:**
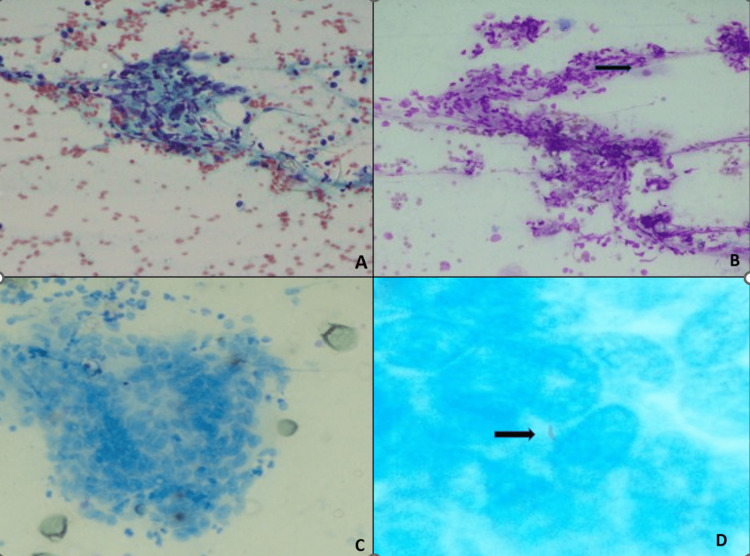
A: The smear shows epithelioid cell granulomas (PAP;200X), B: Wright-Giemsa stain shows epithelioid cell granulomas along with degenerating inflammatory cells and oral squamous cells from the tract (WG;200X), C: Langhans giant cell (ZN;400x), D: Acid-fast bacilli visualized within the giant cell (ZN;1000X)

USG's neck showed multiple enlarged (left) level 2, 3, and 4 lymph nodes (Figure 1). Cartridge-based nucleic acid amplification test (CBNAAT) on fine needle aspirate was negative, and the X-ray chest posteroanterior view was normal. The patient was given anti-TB treatment, and now the patient is disease-free and doing fine.

Case 2

A 35-year-old male presented with complaints of an acute onset of pain and difficulty while deglutition for seven days. It was associated with swelling over the neck and right side of the face. He also has a history of addiction to smoking for the past few years. On oral cavity examination mouth opening was restricted with the right tonsil being inflamed and pushed medially with multiple pus points. The swelling over the neck extended to the right infra-auricular region. The patient had undergone incision and drainage for the right peritonsillar and parapharyngeal abscess (Figure 1). A tracheostomy had to be performed given impending airway compromise.

**Figure 2:**
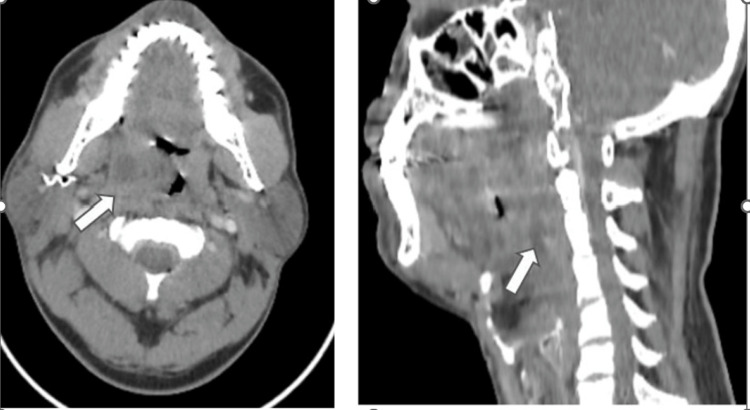
Axial and sagittal CECT of the neck reveals the right tonsillar abscess which is seen extending inferiorly along the posterior pharyngeal wall on the right side (white arrow)

On AFB stain, no acid-fast bacilli were detected. His Hb was 14g/dl, and his TLC was 4420/µl. Contrast-enhanced computed tomography (CECT) of the neck post incision and drainage showed peripherally enhancing loculated collection in the right oropharynx involving the right tonsillar, peritonsillar, and right lateral wall of pharynx causing airway narrowing (Figure 2). Inferiorly there was another peripherally enhancing loculated collection in the right hypopharynx. Hence, the patient underwent an emergency repeat incision and drainage of the residual collection in the peritonsillar space. Pus was sent for bacterial culture sensitivity and CBNAAT, and on the latter *Mycobacterium tuberculosis* (M.Tb) was detected. The patient was given anti-tuberculous treatment, and now the patient is disease free and doing fine.

Case 3

A 22-year-old male reported to the outpatient department for complaints of an evening rise in temperature which was mild for a few weeks. It was accompanied by weight loss and a dry cough. Later he observed swelling in the right tonsillar fossa, and the tonsil was hypertrophied. An intraoral fine needle aspiration was done and 2 ml pus was aspirated. The smears showed acute suppurative inflammation. On CBNAAT, the pus tested positive for M.Tb. The rifampicin resistance result was indeterminate.

**Figure 3:**
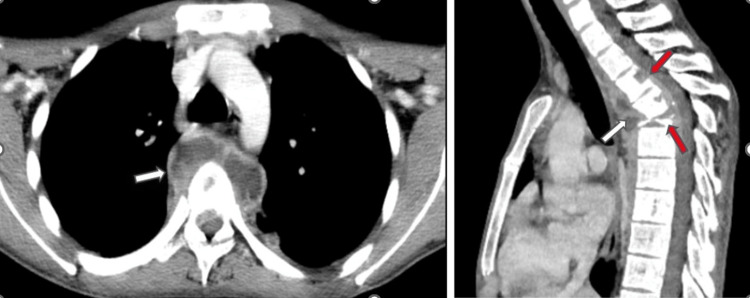
Axial and sagittal of CECT thorax reveal complete wedge collapse of the body of D5 vertebrae (red arrow) resulting in focal kyphotic deformity. Lytic lesions are also noted in the posterior aspect of the body of the D3 and D4 vertebrae (red arrow). Peripherally enhancing abscess (white arrow) is noted in the pre- and paravertebral region (D3 to D6 levels) and extending into the spinal canal causing mild narrowing of the thecal sac

**Figure 4:**
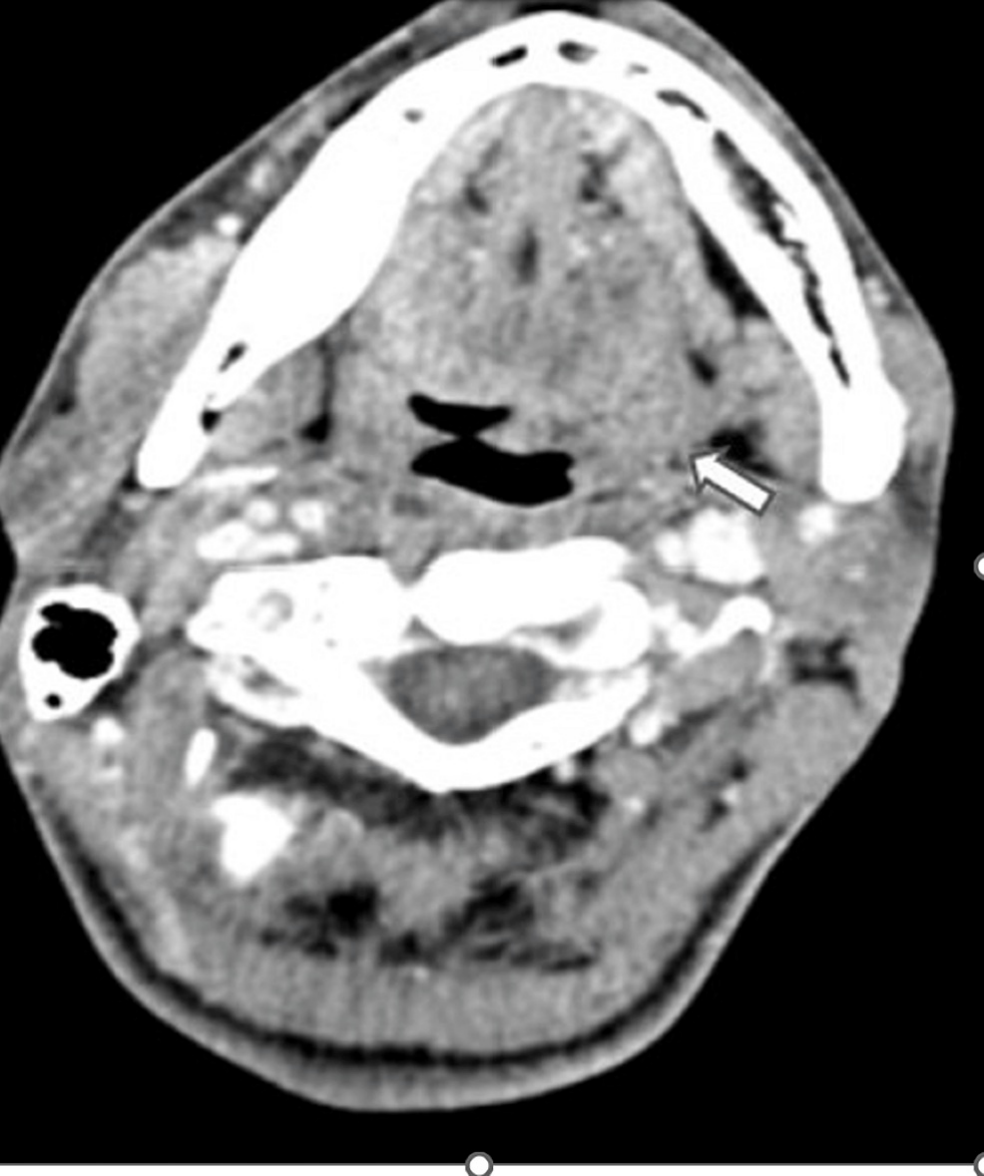
Axial CECT section of the oral cavity reveals bulky and enlarged left tonsillar fossa (white arrow)

The patient had a history of neck pain with restricted backward movement of the neck for a few weeks. Hence, the patient was advised CT scan of the neck and thorax, which revealed a collection in retropharyngeal space from C1 to C5 causing narrowing of the oropharynx and involving the long capitis muscle, suggestive of Pott’s spine (Figures 3-4).

Case 4

**Figure 5:**
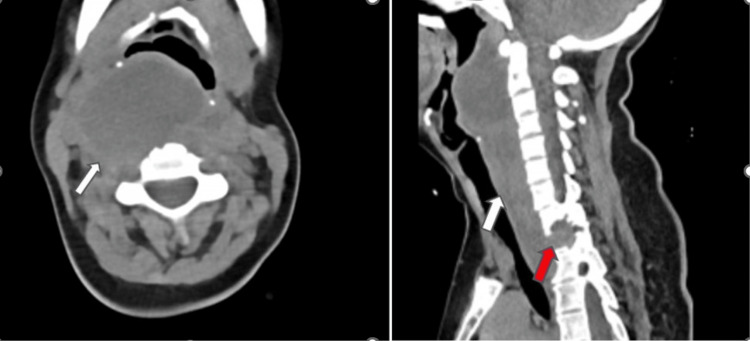
Axial and sagittal non-contrast CT scan of the neck shows a large hypodense collection (white arrow) in the retropharyngeal space extending from the skull base to the D3 vertebral level displacing the oropharynx and esophagus anteriorly. Lytic partial destruction of the lower half of the body of the D1 vertebra and complete destruction of the body of the D2 vertebra is also noted (red arrow)

**Figure 6:**
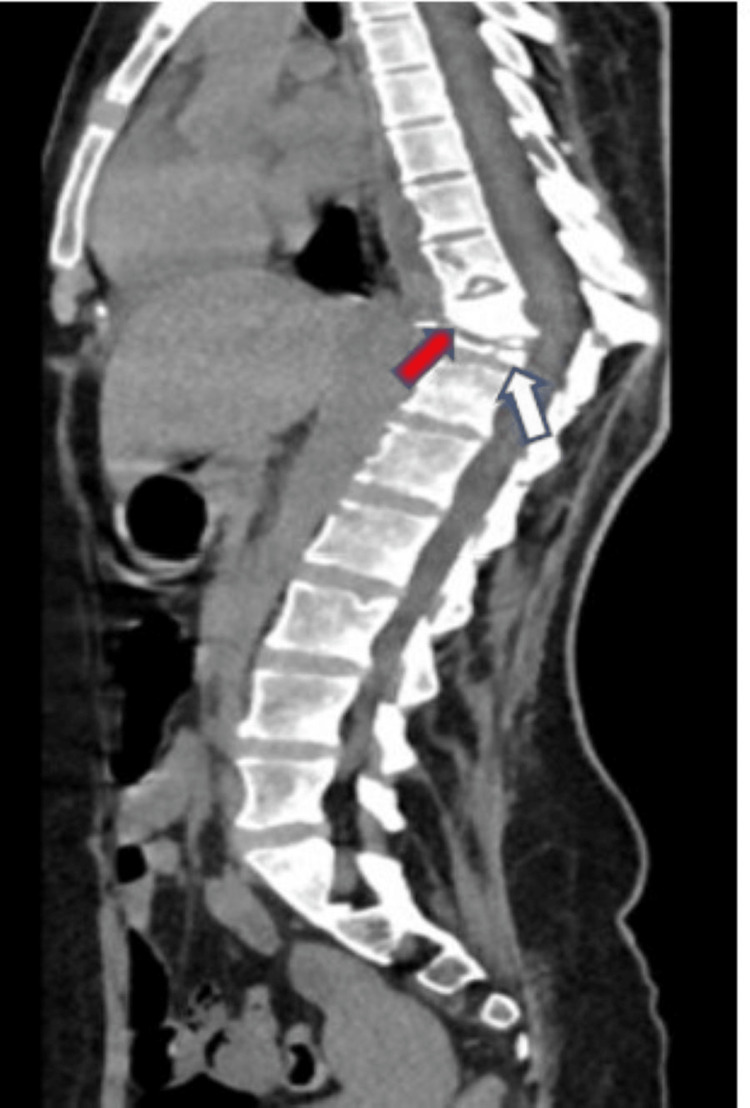
Sagittal non contrast CT of the dorso-lumbar spine reveals lytic destruction complete collapse of the D11 vertebra (white arrow) and partial antero-inferior wedge collapse of the D10 vertebra (red arrow) with bony fragments in the prevertebral soft tissue

A 25-year-old female presented with swelling in the right tonsillar area for one to two months along with pain in the neck and back with restricted movements for a few weeks. She also had an evening rise in temperature. Aspiration was done from the oropharyngeal swelling which revealed a TB abscess on CBNAAT testing as M.Tb was detected. In addition, CBNAAT detected rifampicin resistance. The case was multidrug-resistant TB as resistance to isonicotinic acid hydrazide (INH) and rifampicin was detected on the line probe assay of M.Tb culture. CT scan of the neck and thorax revealed a hypodense collection in retropharyngeal space extending from the skull base to D1, displacing the oropharynx and esophagus anteriorly. There was lytic destruction of D1 and D2 vertebrae and collapse of D10 and D11 vertebrae, suggestive of Pott’s spine (Figures 5-6). This patient was resistant to INH and rifampicin and was treated with bedaquiline, levofloxacin, linezolid, clofazimine, and cycloserine.

## Discussion

India contributes to 23% of the global TB burden. The lung is the most common site of primary TB, and other primary sites are the intestine, tonsil, skin, and rarely nose and genitourinary tract. Primary oral and oropharyngeal TB accounts for 0.05% of the total TB cases in the absence of pulmonary focus [4]. M.tb is an aerobic, acid-fast, nonmotile, non-encapsulated non-spore-forming bacillus with a cell structure that prevents it from phagolysosomal fusion formation within the macrophage [5]. Tonsil is the commonest subsite of the oropharynx followed by the oropharyngeal wall and palate. The upper respiratory tract is somewhat resistant to tuberculous bacilli due to protection provided by thick stratified squamous epithelial lining, oral saprophyte commensals, cleansing action of saliva, immunoglobulin A, and the presence of salivary enzymes. The salivary enzymes with microbicidal action are lysozyme, lactoperoxidase, and lactoferrin. The infection is encountered in patients with poor immunity due to alcoholism, HIV infection, or any breach because of inflammation or trauma to the mucosa, tooth extraction, or poor oro-dental hygiene which may provide a route of entry for M.tb [6].

Spinal tuberculous (Pott’s spine) infections are always secondary to hematogenous spread from the primary focus, and its presentation is varied. It is insidious in onset and progresses slowly with backache as the most common presenting symptom [7]. In the cervical spine, it is seen as a cold abscess which presents as a retropharyngeal abscess. It produces dysphagia, hoarseness, respiratory distress, nerve root compression, and sensory involvement [8].

The oropharyngeal TB patient usually presents with sore throat dysphagia and cervical lymphadenopathy with or without tonsillar enlargement, ulceration, masses, or white patches seen on tonsils. The final diagnosis of oropharyngeal TB is made up of pathological features of caseating necrosis, epithelioid cells, Langhans giant cells, and variable inflammatory cell infiltrate. Techniques such as polymerase chain reaction can be used but should be correlated with cytopathology or histopathology before starting treatment. Among the new generation of automated molecular diagnostic platforms, CBNAAT has the potential to diagnose TB and rifampicin resistance within two hours. Its high sensitivity and less time taking makes it an excellent tool for early diagnosis [9].

TB is classically known as a chronic granulomatous infectious disease with a long course. Though acute form can exist, the pathogenesis of which is incompletely understood, which may be related to epidemiological and genetic host factors. The well-known form of acute TB is the form of miliary TB as acute TB meningitis or acute abdominal obstruction. Critically ill patients may present with acute respiratory distress syndrome, shock, or disseminated intravascular coagulopathy. However, the location and status of the organ involved, the general condition of the patient, and other comorbid illnesses may affect the presentation in the individual patient.

Isolated pharyngeal lesions may be acquired by inhalation with harboring of the disease in the Waldeyer’s ring. The bacilli utilize the lymphohematogenous route to spread and create new infectious foci. Our patients were immunocompetent with no previous history of any contact with TB cases. Patients with a history of addiction to tobacco could be the cause of repeated episodes of inflammation or trauma to the mucosa with poor oro-dental hygiene, which could explain the localization of TB bacilli in the Waldeyer’s ring. Two of our cases were drug-sensitive that is rifampicin and isoniazid sensitive and were treated with anti-tubercular treatment, and the other two were drug-resistant tubercular cases that were rifampicin- and isoniazid-resistant that were treated with bedaquiline, levofloxacin, linezolid, clofazimine, and cycloserine.

During our investigation, we encountered normal ESR, no anemia, and no leukocytosis, with negative sputum and throat swab culture and normal chest X-ray in the initial examinations. Hence, a high index of suspicion is required to diagnose oropharyngeal TB and also to differentiate it from other common causes of unilateral tonsillar enlargement like a peritonsillar abscess, where the pus collects between the superior constrictor and capsule of the tonsil, intratonsillar abscess, where the pus collects within the substance of tonsil due to blockage of the crypt, tonsillolith, where lime salts are deposited to form calculi due to crypt blockage, and tonsillar malignancy, where there is a ulcerative lesion with bleeding and induration.

## Conclusions

Though there is not much-published data on the acute presentation of TB as in our cases of tubercular tonsillitis and tubercular abscess with Pott’s spine, TB should be ruled out in patients having varied extrapulmonary presentations who are not responding to conventional treatments. It can be seen as granulation or growth in the larynx which can mimic carcinoma. In the ear, it is seen as pale granulations with thick ear discharge and hearing loss. In the nose, it can present as a thick mass affecting the anterior nose, with blood-stained nasal discharge, obstruction, pain, or dryness. In all these cases, a definitive diagnosis can be made by biopsy for histopathological diagnosis along with a gene expert test.
